# Dual mobility cups for total hip arthroplasty: tips and tricks

**DOI:** 10.1051/sicotj/2020018

**Published:** 2020-06-17

**Authors:** Thomas Neri, Bertrand Boyer, Cécile Batailler, Antonio Klasan, Sebastien Lustig, Remi Philippot, Frederic Farizon

**Affiliations:** 1 Department of Orthopaedic Surgery, University Hospital Centre of Saint-Étienne avenue Albert Raimond 42000 Saint-Étienne France; 2 EA 7424 – Inter-University Laboratory of Human Movement Science, University Lyon – University Jean Monnet Saint-Étienne avenue Albert Raimond 42000 Saint-Étienne France; 3 INSERM U1059 SAINBIOSE avenue Albert Raimond 42000 Saint-Étienne France; 4 Orthopedic Surgery Department, Croix-Rousse Hospital 103 Grande Rue de la Croix-Rousse 69004 Lyon France; 5 North Shore Hospital 124 Shakespeare Road, Takapuna 0620 Auckland New Zealand

**Keywords:** Total hip arthroplasty, Dual mobility cup, Surgical technique, Tips, Tricks

## Abstract

Since its creation in 1974, the Dual Mobility Cup (DMC) has been gaining in popularity, especially in the past decade. This intensive use could lead to inappropriate use and consequently to an increased complication rate. Compliance with conceptual requirements and surgical techniques will prevent the occurrence of complications that can be wrongly attributed to implants. In this context, we feel that it is essential to share our tips and tricks as well as an overview and an explanation of common errors, based on more than 45 years of clinical and research experience. From basic principles, including indications, implant choice and implant compatibility, to surgical tips, in this article orthopedic surgeons will find a practical overview of DMC in order to use it safely and with confidence.

## Introduction

Total Hip Arthroplasty (THA) is one of the most successful procedures in orthopedic surgery. Among various cup types, liner and head combinations that exist, the Dual Mobility Cup (DMC) was invented in 1974 by Gilles Bousquet in Saint-Étienne [1, 2]. The DMC concept combines Charnley’s low-friction arthroplasty principle [3], in which a small-diameter head articulates with an ultra-high molecular weight polyethylene (UHMWPE) liner, and the McKee-Farrar principle [4], in which a head with similar dimensions to the native femoral head articulates with a cup to increase the stability of the prosthetic joint. This combination is possible because the DMC liner maintains mobility within the metal cup. The three primary goals and advantages of the DMC concept are to increase implant stability, restore nearly physiological hip joint range of motion and to reduce wear [1, 2].

Disparaged for a long time, the DMC concept is in full expansion. This concept has evolved since 1974 with several modifications that have reduced the complication rate, improved implant survival, and expanded its indications [5]. Due to good clinical outcomes and survival rate, indications are becoming broader and are no longer limited to revision surgery. This has allowed the DMC concept to spread beyond France and to become international. Several recent publications reflect the surgical community’s growing interest in this implant [6, 7].

The intensification of the use could lead to inappropriate use and consequently to an increased complication rate. Compliance with conceptual requirements and surgical techniques will prevent the occurrence of complications that can be wrongly attributed to implants. In this context, we felt obliged to share our tips and tricks, based on more than 45 years of clinical and research experience [8–10].

This aim of this paper is therefore to provide an overview of technical and theoretical requirements necessary to achieve a successful DMC implantation. Through tips and tricks, we will present a detailed overview of the use of DMC, including indications, choice, and compatibility of DMC implants, surgical techniques, and management of complications.

## Indications and DMC implants choice

Since the beginning, Gilles Bousquet recommended DMC to all his patients requiring THA, without an age threshold: primary and secondary osteoarthritis, femoral neck fracture, aseptic osteo-necrosis, arthritis sequelae, hip dysplasia, congenital dislocation. DMC were also offered for revision surgery, either for recurrent dislocation or for loosening.

However, out of Saint-Étienne and Lyon, these implants were usually used for revision procedures or in patients with a high dislocation risk. Since that time, due to encouraging results of both stability and longevity, the age limit for DMC was reduced, and the indications were expanded and are no longer limited to revision surgery only [6].

### Prerequisites

On one side, DMC advantages are the decreased dislocation risk and restoration of a full hip joint amplitude [5]. Consequently, the first indication widely recommended for using DMC is for patients that are at a risk of dislocation. This risk depends on the etiology, which is higher in revision surgery, in post-traumatic arthritis or in cases of pathoanatomic changes (dysplasia, congenital luxation, and arthrodesis). It also depends on other patient characteristics (neuromuscular deficits, risk of falling) and activity level. Following primary THA, the dislocation rate with DMC is between 0% and 2% regardless of the DMC generation being used [1, 4, 8, 10–20]. The second primary indication for DMC is a patient requiring a restoration of a high range of motion.

On the other side, the main drawback of DMC is wear of the polyethylene liner [21]. For this reason, the ceramic-ceramic bearing couple is the gold standard in active patients. However, even in these young patients, which represent the worst scenario for wear, results with DMC at more than 20 years of follow-up were comparable to those of MoP bearing studies [22]. At 22 years’ follow-up, the survival rate of the DMC was 77% in patients under 50 years of age (mean age of 41 years [9]). The revisions were performed because of liner wear with 15% aseptic loosening and 10% intra prosthetic dislocation (IPD) when the wear affected the retaining ring. The collection of improvements made to this first generation of implants (new coating, better press-fit fixation, change in the liner design and density, and improved clearance between the liner and cup) have reduced the stress at the interface and thus improved implant survival [5]. Long-term survival of first-generation implants, which was already comparable to other bearings, is now close to 100% in the medium term. These promising results must be confirmed by long-term studies.

In any case, the philosophy inherent in the concept of DMC tends toward the restitution of an ideal anatomy, both in terms of dimensions (cup diameter) and positioning (center of rotation). This implies limiting the widening of the cup diameter (jumbo cup type) as far as possible and compromises moving the center of rotation, in order to avoid impingement or premature wear. In revision surgery, it is advisable to keep the dimensions of the revised cup as large as possible for the new revision cup. Bone defects will be filled by bone grafting (allograft, autograft, substitute) or trabecular metal. Our current practice matches Gilles Bousquet’s preference for grafting, allowing for reconstitution of the bone stock around the implants, allowing the chosen metal framework to be fixed at a distance from the revision zone to allow for a good reconstruction.

### DMC in the setting of primary surgery

Press-fit only implants allow adaptation to normal anatomy with adequate bone quality. In this condition, macrostructures and combined titanium and hydroxyapatite coating ensure good primary and secondary fixation (Table 1) [8].

**Table 1:** Issues and their solutions regarding implant choice.

Issues	Solutions
Insufficient bone quality or non-hemispheric acetabulum	Tripod DMC or even a DMC with a hook
Significant acetabular defect	Reinforcement cage + cemented DMC
Extensive bony acetabular defect	Custom reinforcement cage + cemented DMC
Stick to the patient anatomy in complex revision	• Inner diameter of the reinforcement cage = outer diameter of the initial cup.
	• Cemented DMC diameter = initial cup diameter −4 to 6 mm (thickness of cement).

In the case of dysplasia or congenital dislocation, we recommend the use of an implant with additional fixation properties. Our preference is to use tripod implants that reinforce press-fit fixation in all three planes. Small diameter tripod implants should be used in these indications to preserve bone stock and maximize bone coverage around the implant. These implants can also be used to secure an acetabular bone graft. Although they are larger and require more exposure time, tripod implants with obturator hooks and screwed flanges are also an option in these types of cases.

### DMC in the setting of revision surgery

**Figure 1:**
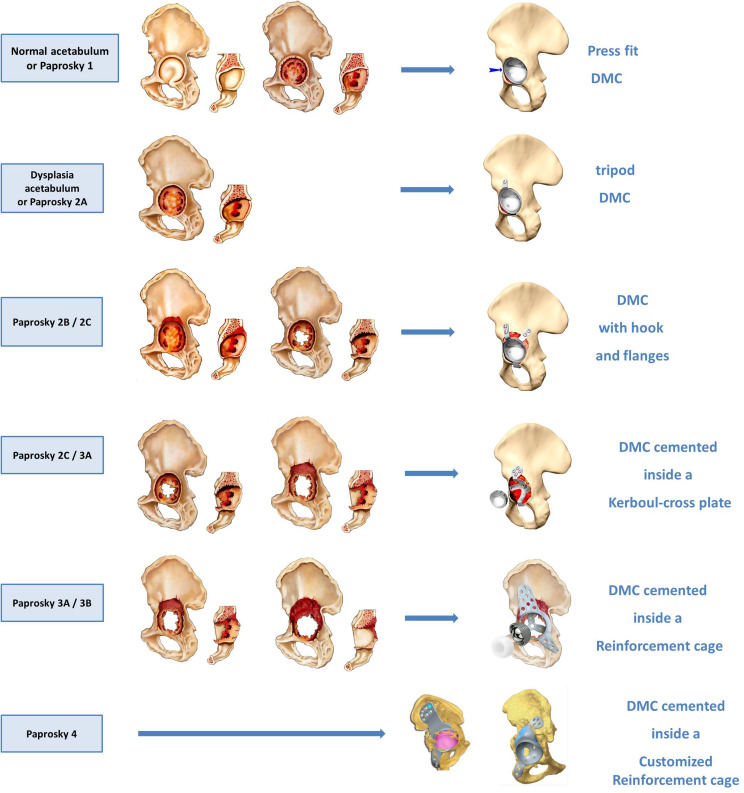
DMC choice regarding Paprosky classification (reprinted with SERF^®^ permission).

Once the initial implant has been removed and the periacetabular area is exposed, the evaluation of bone defects allows for orientation toward the type of implant required. Usually, we used the Paprosky classification for planning further steps (Figure 1).

If there are no defects (Paprosky 1A) and the bone quality is good, the press-fit only implant is possible. However, we advise to maximize the primary fixation by a tripod implant if the slightest doubt occurs. This is an almost systematic attitude in our experience, as this implant does not require any additional bone preparation and the time necessary for the positioning of the pegs and the screw is minimal with regard to the outcome achieved.

For 2A stage, the use of a tripod cup is always recommended [23]. For lesions 2B (walls) or 2C (back), the implant with obturator hook and screwed flanges allows a third additional means of fixation to be added, while maintaining the press-fit and tripod fixation [24]. The addition of graft (or trabecular metal) is thus facilitated.

For more extensive 2C and 3A stages, the addition of a metal reinforcement allows fixation in healthy bone. We use a Kerboull cross-plate. A special DMC is cemented into this. Its dimensions should be close to the cup initially planned, and it is these dimensions that guide the choice of the external diameter of the cross-plate and not the other way round [25–27]. However, surgeons have to keep in mind the thickness of the cement required to fix the DMC. A minimum cement thickness must be respected, to avoid galvanic coupling (cup-structure contact) but also to maximize cup fixation. Two-to-three-mm cement thickness seems sufficient, resulting in a cemented cup diameter of 4 or 6 mm less. We recommend ideally matching the internal diameter of the cross-plate to be chosen with the external diameter of the initial implant, as this seems to be a good compromise between bone reconstruction, mobility and stability.

However, the Kerboull cross-plate is not sufficient for internal fixation in stages 3B and 3C, in which case a Burch-Schneider-type reinforcement cage can be used [27].

For stage IV, pelvic discontinuity in most cases requires a custom-made tri-flange reinforcement cage, allowing farther fixation while respecting the patient’s anatomy. The surgeon will choose whether he prefers to fill the loss of substance with metal, if possible trabecular, or allograft (massive or, at our preference, fragmented).

## Implant compatibility for DMC

Like for any orthopedic implant, the use of MDC requires guidelines. There are rules that should be followed in order to avoid complications. In general, implants designed specifically for DMC should always be favored. Schematically, the DMC system is composed of a cup, a mobile liner, and a femoral head impacted on the femoral stem (Table 2).

**Table 2:** Tips and tricks for implant compatibility

Cup	The cup should be specifically designed for a dual mobility system.
Liner	The liner used must be from the same manufacturer as the cup. The liner and cup are an inseparable combination.
	The size of the liner must be in accordance with the size of the cup; according to the manufacturer’s recommendations.
	UHMWPE or XLPE liners may be used. Please note that no long-term studies are available on the evaluation of the XLPE in DMC.
Femoral head	Heads with a diameter of 22 mm are to be favored. 28 mm diameter heads should not be used with cups smaller than 54 mm.
Femoral stem	The femoral stem used should have a thin polished neck that is trapezoid, elliptical or circular in shape.

### Cup

The shape is usually cylindrical-spherical. This shape increases the jump distance and thus reduces the risk of dislocation. Most DMCs are symmetrical. The DMC was created with a cementless fixation rationale in mind. However, cemented models do exist, most often in combination with a metal reinforcement cage. DMC cemented into the native acetabulum remains possible. The DMC cups available are either made of Cobalt-Chrome-Molybdenum (CoCrMo) or 316L stainless steel.

#### Cup-liner

The first rule is to use the correct liner for the cup being used, from the same manufacturer, designed specifically to be used with the cup. The liner and the cup are inseparable, in order to respect the clearance between the cup and the liner. Schematically, the liner has an external diameter slightly smaller than the internal diameter of the cup. This difference, or clearance, is already calculated by the manufacturer. It is absolutely necessary not to use a larger liner (risk of blockage) or a smaller liner (risk of early wear). The clearances have been altered to avoid jamming of the inner and outer surfaces of the liner.

#### Liner density

Since the first PEs, the liners are now denser (UHMWPE), reducing wear complications. The classical PE is UHMWPE. DM liners made of cross-linked polyethylene (XLPE) have been recently introduced. The benefits of this latest modification are still being debated. These new liners raise the crucial question of behavior at the retention collar at the time of impaction (femoral head into the liner). This leads to many questions that remain unresolved to this day: risk of microfractures, adaptation of the design of the collars to XLPE, etc. [28, 29]

#### Liner-femoral head

The DMC system has been designed to be used with 22 mm heads in order to respect the logic of Charnley’s theory (smaller diameter, less wear). However, 28-mm heads can nowadays be used, provided they are used in large liners to avoid insufficient PE thickness in the liner. In order to guarantee a circumferential thickness of at least 10 mm PE, we recommend using 28-mm diameter heads only for liner sizes larger than 54 mm.

The design of the liners has changed considerably since the introduction of the first DMC. The chamfer and retention mechanisms were redesigned to reduce wear in the retaining ring. This resulted in a significant reduction in the occurrence of intraprosthetic dislocation (IPD) [2, 28, 30].

#### Femoral head

The composition of the heads can be either 316L stainless steel, CrCo, or ceramic. Skirted head systems are not recommended. These systems, by increasing the diameter of the neck, will promote contact between the femoral neck and the retention system of the liner and can lead to an IPD.

#### Femoral stem

The first generations of DMC were associated with femoral stems with wide, rough necks. With a contact between the neck and ring, this combination led to the occurrence of a specific complication of DMC: the IPD [2, 28, 30]. Since the change to a thin polished neck that is trapezoid, elliptical, or circular in shape, no IPD has been reported. In summary, combination DMC and liner are inseparable and must be issued by the same manufacturer. The liner and femoral stem combination may be from a different manufacturer, but the stem must have a neck compatible with the DMC system.

## Intra operative tips and tricks

### Pre-operative planning

Like a classic THA, DMC is not made to compensate for deficiencies in planning. The basic principles of hip arthroplasty must be respected. Restoration of the patient anatomy remains the key to success for good clinical outcomes and to avoid complications. Among them, restoration of the hip center and the femoral offset, as well as the position of the femoral osteotomy in primary surgery are crucial points on which planning should be based.

### Posterior approach

Historically, DMC has been developed for the posterior approach. In Saint-Étienne department, all patients were operated using a postero-lateral approach, developed initially by Moore. Although the posterior approach is associated with a higher dislocation rate, the use of DMC has resulted in dislocation rates comparable to implants placed through a direct anterior approach. In large studies with more than 20 years’ follow-up conducted in our department, no dislocations occurred in spite of exclusive use of the posterior approach [10, 11]. The main advantage is the ease of access and excellent exposure, which allows good positioning of the implants which is necessary to minimize complications. Disadvantages are the risk of damage to the sciatic nerve, which is extremely rare, and a slower recovery time due to the surgical dissection of external rotators of the hip.

Some tips and tricks can facilitate exposure, bony preparation, implant positioning, and intra-operative testing (Table 3).

**Table 3:** Tips and tricks for posterior approach.

Soft tissue management	• We encourage a minimally-invasive approach with preservation of piriformis tendon.
	• No specific recommendations for closing and reconstructing of the joint capsule and external rotator muscles.
	• Keep the transverse acetabular ligament intact to determine the acetabulum version.
Acetabulum preparation	• Requires a good acetabular exposure.
	• Start by reaming vertically with a small diameter reamer in order to remove all the osteophytes around the acetabular fossae.
	• The inferior edge of the reamer is then placed in line with the transverse acetabular ligament to respect anatomic anteversion.
	• Try to use smallest cup size that will allow a good primary fixation.
Cup positioning	• Keep a sufficient anteversion to avoid psoas impingement and decrease the risk of dislocation.
	• A supero-posterior bone coverage defect of the cup is often observed and has no consequences.
Intraoperative testing	• For stability tests, the hip is flexed at 90° and an internal rotation is gradually applied. If dislocation occurs at more than 45°, the THA is considered stable.
	• For length and femoral offset parameters, the piston sign observed with knee fully extended has to disappear when the knee is flexed at 90° of flexion.

#### Soft tissue management

The first question often asked is: “Should the tendon of the piriformis muscle be dissected or preserved? This question is related the approach itself rather than the use of DMC. Following the trend of minimally invasive surgery, we keep it intact in primary surgery. Specific instruments have been developed for that, allowing for a reduction of both the size of the scar and the soft tissue damage. These include curved retractors and reamers.

The second question is: “Is it necessary to reconstruct the joint capsule, as well as the external rotators when using a DMC?”. Some surgeons advocate that it increases external rotation force and reduces the dislocation rate. Others, including Gilles Bousquet, leave the joint open and suture only the fascial layer and the gluteus maximus aponeurosis. To our knowledge, the literature does not answer this question. It depends mainly on the surgical training habits.

#### Acetabulum preparation

**Figure 2:**
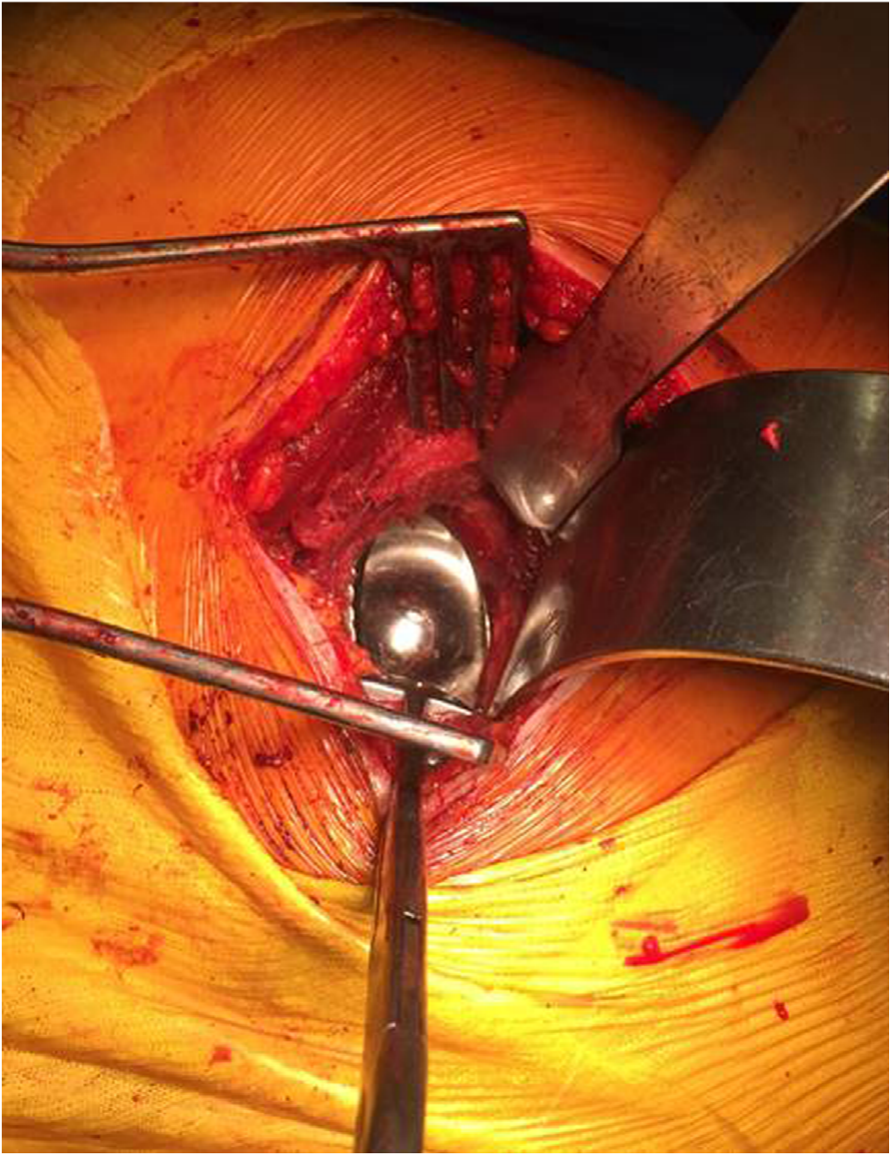
Posterior approach: exposure and cup positioning. One anterior Hohmann retractor is positioned in the anterior horn and a self-retainer, taking out the capsule, is placed close to the posterior horn. The cup is positing in line to the transverse acetabular ligament to restore the anatomic anteversion.

For this step, the fundamental prerequisite is a good exposure of the acetabulum. An anterior retractor is placed on the anterior horn after removal of the acetabular labrum. In order to expose the posterior part, we use a “capsule self-retainer” taking out the capsule, close to the posterior horn (Figure 2).

Especially in severe osteoarthritis, we start the reaming around the acetabular fossa. It allows for restoration of the anatomic hip center and prevents proximal reaming. For this step, we use a small reamer and start reaming vertically, or perpendicular to the horizontal axis.

After this first step, we ream directly into the anatomic position. We keep the transverse acetabular ligament intact to determine the anatomic anteversion of the acetabulum. The inferior edge of the reamer is placed in line with this ligament.

As previously explained, we try to use press fit DMC as much as possible. The fundamental principle of this is to use the smallest cup size possible that at the same time allows a good primary fixation. Usually, for primary osteoarthitis we try to use a cup size that is similar to the femoral head diameter, or slightly larger (e.g 45-mm or 47-mm DMC for femoral head of 45 mm). For neck of femur fractures, with lower bone density, the targeted size is one or two mm bigger than the femoral head dimeter. If the primary stability is not achieved with the trial cup of the proposed size, we prefer to use a tripod cup in order to avoid impingement risk which exists with big cup sizes.

#### Cup positioning

Sufficient anteversion is required to avoid psoas impingement and to increase hip stability (Figure 2). The anatomical landmarks used are: the transverse acetabular ligament to control acetabular depth, height and version, anterior and posterior horns as secondary guides for the version and the acetabular roof for controlling of the inclination. In some cases, even in good cup positioning, there is a bone defect in the superior-posterior aspect of the acetabulum. This has no consequence and should not lead to a change in cup positioning.

#### Intraoperative testing

With femoral and cup trial implants, we perform dynamic tests in order to test the stability of the hip, to rule out any risk of impingement and to verify the good restoration of hip center.

For stability tests, the hip is flexed at 90° and an internal rotation is gradually applied. If dislocation occurs at more than 45°, the THA is considered stable.

**Figure 3:**
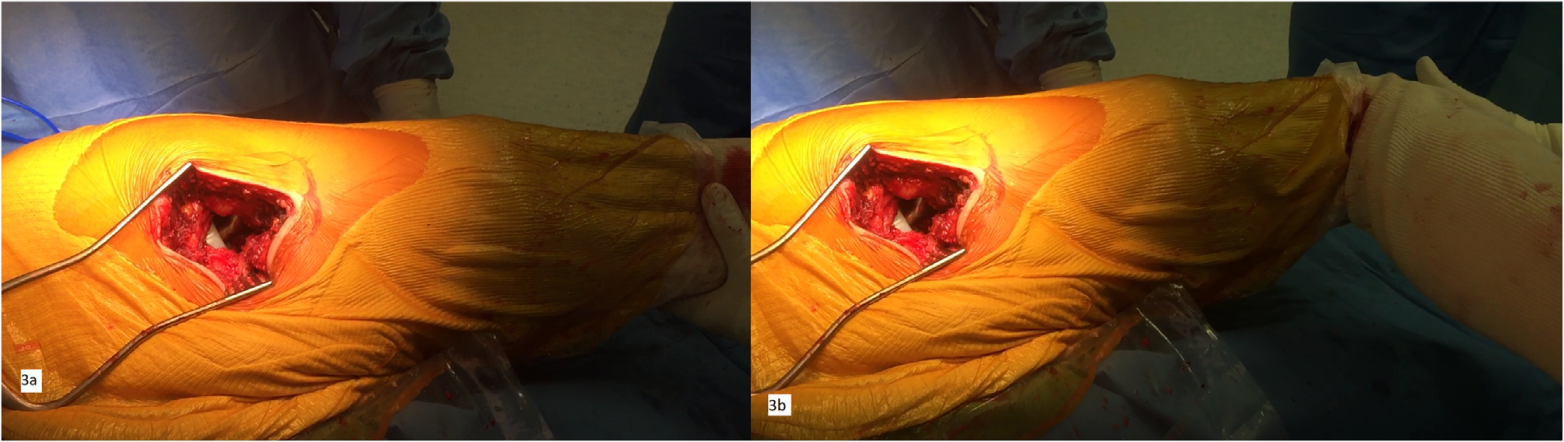
Intra-operative DMC testing with the posterior approach. (a) Test with the knee fully extended showing the “piston sign”. (b) Test with the knee flexed at 90° of flexion showing the disappearance of the “piston sign”.

For length and femoral offset parameters, our main test is based on the evaluation of soft tissue tension. First, with the hip and knee in extension (hamstrings stiffened), traction is applied in the axis of the limb (Figure 3a). The test is considered good if there is a movement, such as a piston in a cylinder, between the head, the liner and the cup. Then the same test is repeated with the knee bent at 90° (hamstrings relaxed). This piston effect should disappear (Figure 3b).

### Anterior approach

The direct anterior approach (DAA) is considered to have a lower dislocation rate due to muscle and posterior capsule preservation [31]. Sariali has described a revision rate for instability of 0.11% on 1764 THA by DAA [32]. However, since the development of the minimally invasive posterior approach (MPA), the differences in terms of stability are less obvious; however, complications remain possible [33]. Some indications of DMC via DAA can be interesting. Few studies have reported the results of a dual mobility cup (DMC) via DAA, which combines a technically demanding approach with a cylindrical-spherical cup [34]. The risk of complications is similar to the other approaches. Some tips and tricks can help avoid main difficulties (Table 4).

**Table 4:** Tips and tricks for anterior approach

Soft tissue management	• Capsulectomy must be performed.
	• The reflected head of rectus femoris can be released at the superolateral part of the acetabulum.
	• The anterior retractor should be positioned in the tear drop and not in the upper part of the anterior horn.
Acetabulum preparation	• A femoral release after the femoral cut is key in improving acetabular exposure.
	• A slight traction of the leg can open the space to pass the reamer.
Cup positioning	• Keep a sufficient anteversion to avoid psoas impingement.
	• Intra-operative fluoroscopy confirms appropriate positioning of implants.
Intraoperative testing	• The use of a standard Table allows an efficient intraoperative test of the hip, similar to a posterior approach.

#### Soft tissue management

**Figure 4:**
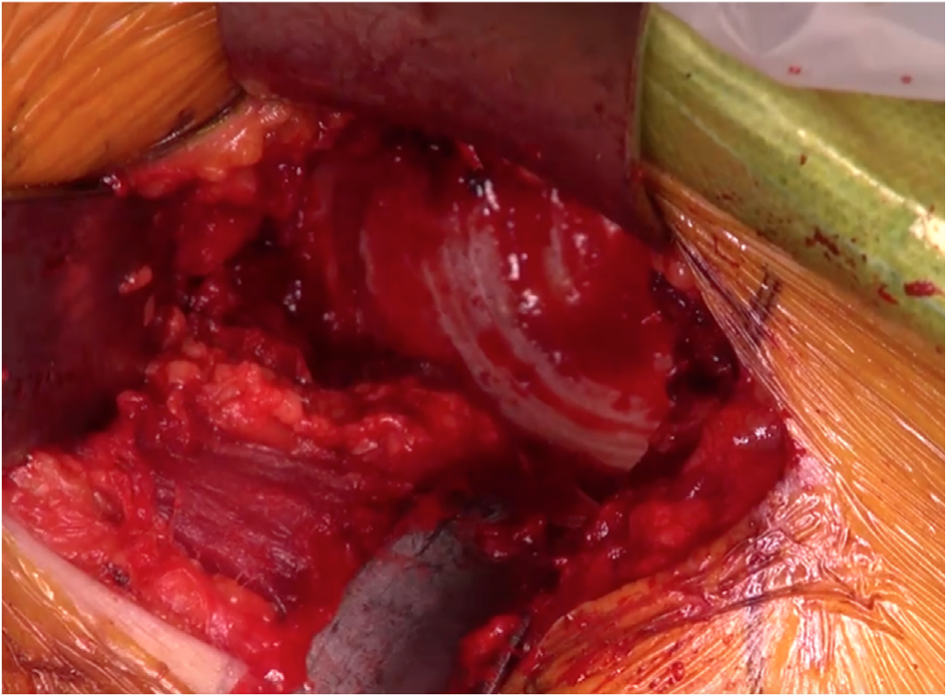
Two Hohmann retractors are positioned in the tear drop and on the posterior horn.

DAA is performed according to the “Hueter Gaine” anterior approach [35], in supine position with a standard orthopedic table or with a traction table. The approach is typical until the acetabulum is reached. Some tips can facilitate the acetabular exposure and preparation. We recommend a capsulectomy. To improve the acetabular exposure, the reflected head of rectus femoris can be released at the superolateral part of the acetabulum. Two Hohmann retractors are positioned on the anterior and posterior horns. The anterior retractor should be positioned in the tear drop and not in the upper part of the anterior horn (Figure 4). This retractor could injure the psoas tendon if it is too high. The anterior retractor must be held with control to avoid a fracture of the anterior horn. To avoid injuries of the soft tissue or the femur, the reamer must be positioned in the acetabulum by sliding on the posterior retractor.

The soft tissue is usually not injured by the reamer arm, because the acetabulum is in the axis of the scar. Only with a bikini scar, the surgeon must protect the skin to avoid injuries.

#### Acetabulum preparation

**Figure 5:**
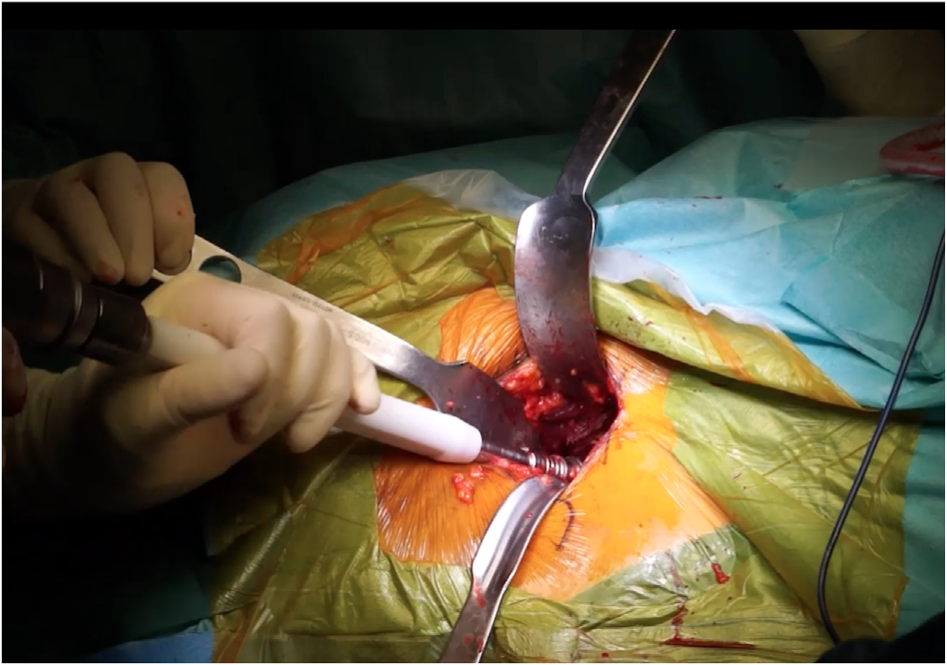
For the direct anterior approach, the straight cup reamer is in the axis of the scar.

**Figure 6:**
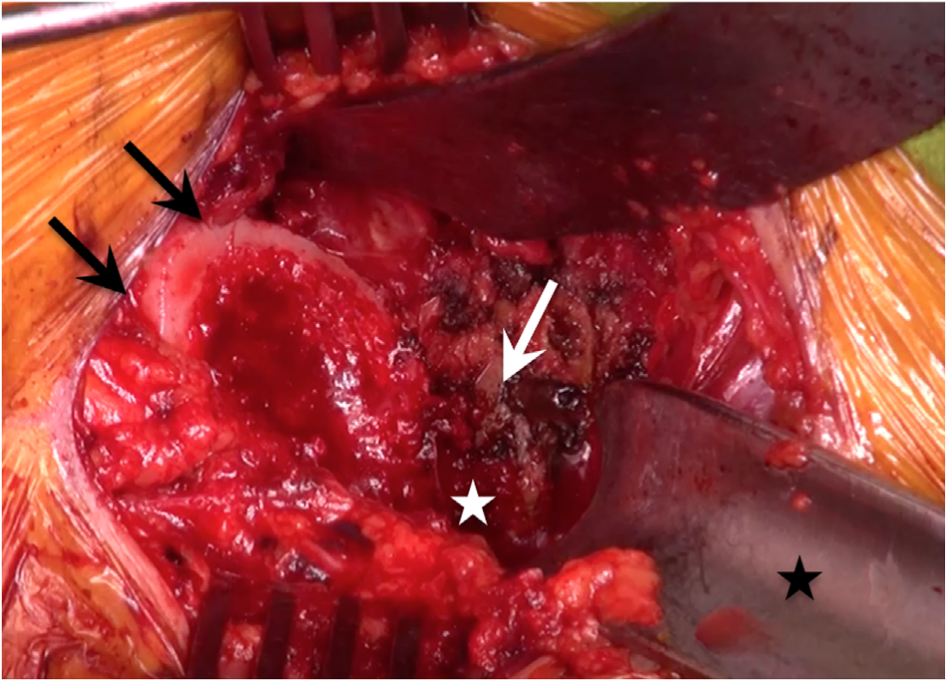
After the femoral osteotomy, it is important to perform a femoral release, with a posterior (white arrow) and lateral capsulotomy (white star) around the greater trochanter (the black arrows show the calcar).

In DAA, the reamer is in the axis of the scar, allowing a lower abduction and an appropriate anteversion of the cup without specific instrumentation, compared to PLA. The preparation follows the same rules as in the posterior approach, with a standard straight cup reamer and impactor (Figure 5). The femur is occasionally in front of the acetabulum and impedes the way for the reamer. That is why the posterior retractor must be positioned well to push the femoral neck back. Two tricks can improve the preparation of the acetabulum. Firstly, after the femoral osteotomy, it is important to perform a femoral release, with a posterior and lateral capsulotomy around the greater trochanter (Figure 6). Secondly, a slight traction of the leg with external rotation by the assistant can open the space in front of the acetabulum. The depth of the reaming is assessed as usual.

#### Cup positioning

Supine position in DAA changes the three-dimensional orientation of the acetabulum relative to the surgeon and needs to be taken into consideration when transitioning from the posterior approach. There are two major advantages of the supine position: it creates less alteration of the pelvic orientation than the lateral decubitus position and allows intra-operative fluoroscopy.

The hemispheric characteristic of DMCs can cause difficulties during the implant positioning. A good exposure of the bone landmarks is key. Following our experience with psoas impingement on the large rim of the DMC, we have been careful to keep sufficient anteversion, maintaining the mean anteversion equal or greater than 25°. The acetabular cup is placed manually according to the anatomical landmarks: the transverse acetabular ligament is used to control acetabular depth, height and version, the inclination is assessed by orientating the cup flush in line with the roof. The anterior and posterior horns help with the anteversion.

**Figure 7:**
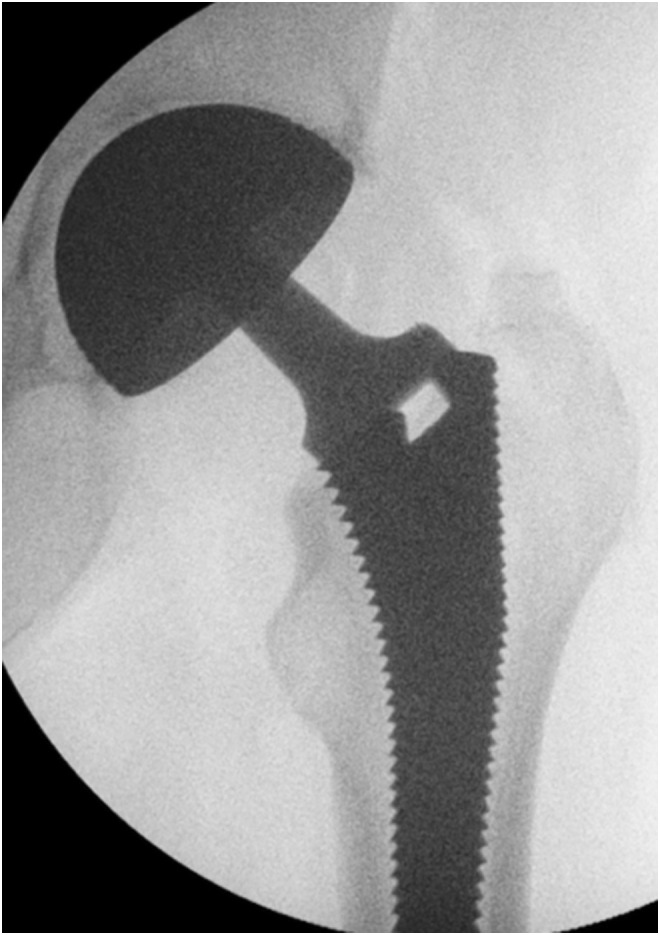
Intra-operative fluoroscopy is used to confirm appropriate positioning of implants.

Intra-operative fluoroscopy is used to confirm appropriate positioning of implants (Figure 7).

#### Intraoperative testing

**Figure 8:**
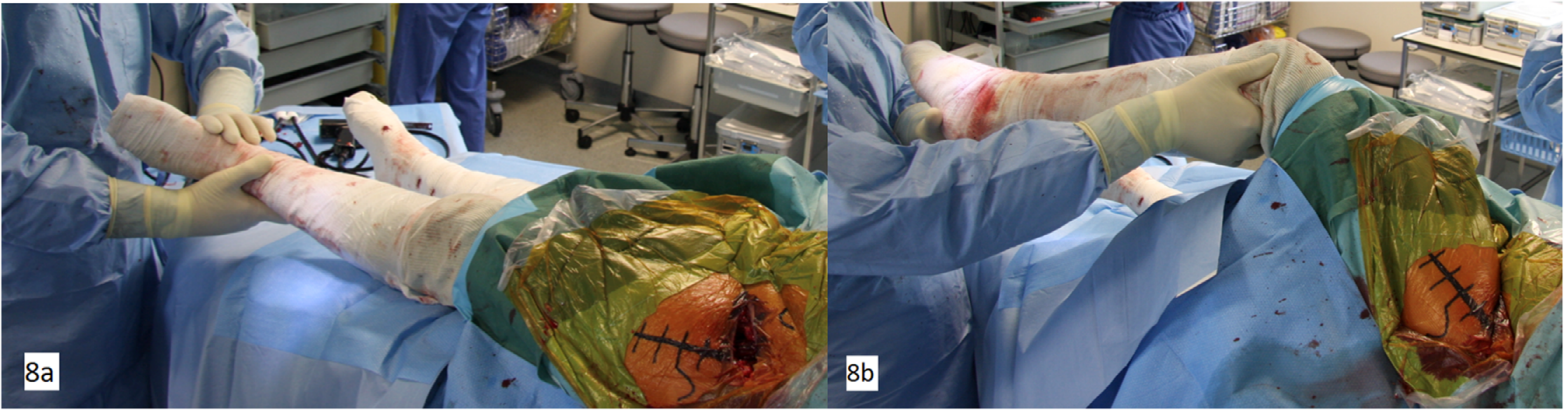
The surgeon can assess the anterior stability in extension and external rotation (a), the range of motion and an eventual impingement (b).

The use of a standard table allows performing an efficient intraoperative test of the hip, similar to a posterior approach. The surgeon can assess the anterior stability in all amplitudes, the range of motion, and potential impingement with the greater trochanter or with the ischium (Figure 8). The anterior stability of the hip must be tested in hyperextension and external rotation.

## DMC complications management

DMC is not without complications. Possible complications include dislocation, loosening, intraprosthetic dislocation, infection, and impingement (Table 5).

**Table 5:** Tips and tricks for DMC complications management.

Issues	Solutions
DMC THA dislocation	Reduction under General Anaesthesia ± neuromuscular relaxation
Two consecutive dislocations or major deficit of the gluteal muscles	Removable above-knee hip spica cast for one month
Instability despite above-knee hip spica cast	Whiteside transfer
Traumatic intraprosthetic Dislocation	Surgical revision with liner exchange
Early infection	Surgical revision for DAIR with head+liner replacement
Avoid loosening	Respect the patient’s anatomy

### Dislocation

Although the rate of dislocation is very low or even nil in primary DMC DHA, the etiologies associated with gluteal muscular deficits (mainly revision and tumor surgery) have shown a non-zero rate of dislocation [23, 36]. It seems to us essential to know the specific modalities for the reduction of a DMC THA dislocation. This reduction must be performed under general anesthesia while the patient is also neuromuscularly relaxed, as reduction is generally more difficult than for a conventional cup because of the size of the liner. The contact between the liner and the cup during the reduction maneuver can cause a decapsulation of the liner, which we call “traumatic” intraprosthetic dislocation [37–43]. This anti-dislocation device can turn into an anti-reduction device in special circumstances (anterior approach, revision with cage), sometimes requiring an open reduction. In the extremely rare case of early recurrence of a dislocation, we recommend the use of a removable above-knee hip spica cast for one month to allow the soft tissues to heal and to aid hip stability. We also recommend this one-month cast in case of gluteal muscle dysfunction (tumor surgery or revision with trochanter defect). The gluteus medius muscle is essential to the stability of the hip. For the same reason, the muscle transfer described by Whiteside seems to give excellent results in extreme cases (megaprothesis, paraplegic patient) [44].

### Infection

Concerning infection, the rates associated with DMC are equivalent to the rates of other hip arthroplasty concepts. However, it should be noted that the population with the highest proportion of DMC also happens to be the population most at risk of dislocation, i.e. the elderly population, traumatic etiology and revision, which are populations that are at a higher risk of infection. In the case of early infection, Debridement, Antibiotics and Implant Retention (DAIR) is relatively straightforward with a DMC [45]. After synovectomy and lavage, a change of the modular components is performed. After dislocation of the hip and uncoupling of the Morse taper from the stem, a new head-liner pair, prepared on the “clean” operating table is implanted.

### Aseptic loosening

Loosening rates are not different from other concepts in the literature, with wear rates closer to cemented Charnley type metal-PE pairs than uncemented metal-PE pairs [46–54]. The cup survival rate for the historical series was greater than 90% at 25 years [10]. This rate seems to be higher for recent implants, using cups with macrostructures and titanium-hydroxyapatite coating, which according to some authors constitute the second generation of dual mobility implants [8, 14, 15, 17, 55]. DMC revision implants also do not have a higher loosening rate than other series, with competitive dislocation rates [23, 56].

### Intraprosthetic dislocation

IPD, caused by wear of the retention collar, is the specific complication of DMC [28, 57, 58]. In historical series, this rate was around 4% and occurred before 10 years since the index operation [1, 10, 57, 58]. The recent series no longer include this complication, with more than 15 years of experience. Modifications to the design of the collar, improvement in the properties of the polyethylene, combined with a compatible DM-stem association (thin polished neck that is trapezoid, elliptical or circular in shape) have probably led to this probable disappearance [28]. However, one should always keen in mind the traumatic IPD.

Surgical treatment is necessary as soon as the diagnosis is made. For traumatic IPD, an open reduction with a liner exchange is required. For IDP due to liner wear, at the minimum, the acetabular cup and the bearing (femoral head and liner) must be exchanged, in combination with a synovectomy [59, 60].

### Impingement

With regard to the impingement, the mobility of the liner prevents it from causing rigid contact with the soft tissues. Impingement can only occur between the cup and the soft tissue, mainly the psoas muscle, or between the neck and the cup.

As DMC requires a thin neck for collar retention purposes, the neck-cup impingement seems anecdotal to us, unless the femoral stem is poorly positioned. This is unlikely to have consequences, as Dual Mobility is not subject to a restricted Lewinnek safe zone. To avoid contact with the psoas, the same instructions must be followed as with a conventional cup, i.e. avoid anterior overhang and oversizing [5, 60].

## Conclusion

This paper provides advice and recommendations for orthopedic surgeons using DMC. The forty years of history since the introduction of DMC have demonstrated its contribution to hip prosthetic surgery, and they have also allowed for refinement of the appropriate indications for its use and the procedure to follow in the event of a complication. With recommendations and evidence from the literature, combined with our experience, these guidelines will allow for a surgeon, not confident with the use of DMC, to safely start using it.

## Conflict of interest

RP and FF receive royalties from SERF (Décines, France).

SL declares the following conflicts of interest: consulting (Stryker), institutional research support to Corin and Amplitude.

## References

[1] Farizon F, de Lavison R, Azoulai JJ, Bousquet G (1998) Results with a cementless alumina-coated cup with dual mobility. A twelve-year follow-up study. Int Orthop 22, 219–224. 9795807 10.1007/s002640050246 PMC3619611

[2] Noyer D, Caton JH (2017) Once upon a time. Dual mobility: history. Int Orthop 41, 611–618. 27990600 10.1007/s00264-016-3361-6

[3] Charnley J, Kamangar A, Longfield MD (1969) The optimum size of prosthetic heads in relation to the wear of plastic sockets in total replacement of the hip. Med Biol Eng 7, 31–39. 5771305 10.1007/BF02474667

[4] Caton JH, Prudhon JL, Ferreira A, et al. (2014) A comparative and retrospective study of three hundred and twenty primary Charnley type hip replacements with a minimum follow up of ten years to assess whether a dual mobility cup has a decreased dislocation risk. Int Orthop 38, 1125–1129. 24737147 10.1007/s00264-014-2313-2 PMC4037498

[5] Neri T, Philippot R, Klasan A, et al. (2018) Dual mobility acetabular cups for total hip arthroplasty: advantages and drawbacks. Expert Rev Med Devices 15, 835–845. 30345834 10.1080/17434440.2018.1538781

[6] Batailler C, Fary C, Verdier R, et al. (2017) The evolution of outcomes and indications for the dual-mobility cup: a systematic review. Int Orthop 41, 645–659. 28004142 10.1007/s00264-016-3377-y

[7] Jonker RC, van Beers LWAH, van der Wal BCH, et al. (2020) Can dual mobility cups prevent dislocation without increasing revision rates in primary total hip arthroplasty? A systematic review. Orthop Traumatol Surg Res 106, 509–517. 32278733 10.1016/j.otsr.2019.12.019

[8] Laurendon L, Philippot R, Neri T, et al. (2018) Ten-year clinical and radiological outcomes of 100 total hip arthroplasty cases with a modern cementless dual mobility cup. Surg Technol Int 32, 331–336. 29689589

[9] Philippot R, Neri T, Boyer B, et al. (2017) Bousquet dual mobility socket for patient under fifty years old. More than twenty year follow-up of one hundred and thirty one hips. Int Orthop 41, 589–594. 28091769 10.1007/s00264-016-3385-y

[10] Neri T, Philippot R, Farizon F, Boyer B (2017) Results of primary total hip replacement with first generation Bousquet dual mobility socket with more than twenty five years follow up. About a series of two hundred and twelve hips. Int Orthop 41, 557–561. 28025659 10.1007/s00264-016-3373-2

[11] Boyer B, Philippot R, Geringer J, Farizon F (2012) Primary total hip arthroplasty with dual mobility socket to prevent dislocation: a 22-year follow-up of 240 hips. Int Orthop 36, 511–518. 21698430 10.1007/s00264-011-1289-4 PMC3291786

[12] Leclercq S, Benoit JY, de Rosa JP, et al. (2013) Evora^®^ chromium-cobalt dual mobility socket: results at a minimum 10 years’ follow-up. Orthop Traumatol Surg Res 99, 923–928. 24176671 10.1016/j.otsr.2013.07.017

[13] Prudhon J-L, Ferreira A, Verdier R (2013) Dual mobility cup: dislocation rate and survivorship at ten years of follow-up. Int Orthop 37, 2345–2350. 24026216 10.1007/s00264-013-2067-2 PMC3843189

[14] Vermersch T, Viste A, Desmarchelier R, Fessy M-H (2015) Prospective longitudinal study of one hundred patients with total hip arthroplasty using a second-generation cementless dual-mobility cup. Int Orthop 39, 2097–2101. 26346372 10.1007/s00264-015-2985-2

[15] Ferreira A, Prudhon J-L, Verdier R, et al. (2017) Contemporary dual-mobility cup regional and private register: methodology and results. Int Orthop 41, 439–445. 28197703 10.1007/s00264-017-3405-6

[16] Vielpeau C, Lebel B, Ardouin L, et al. (2011) The dual mobility socket concept: experience with 668 cases. Int Orthop 35, 225–230. 21184223 10.1007/s00264-010-1156-8 PMC3032118

[17] Epinette J-A (2015) Clinical outcomes, survivorship and adverse events with mobile-bearings versus fixed-bearings in hip arthroplasty-a prospective comparative cohort study of 143 ADM versus 130 trident cups at 2 to 6-year follow-up. J Arthroplasty 30, 241–248. 25449593 10.1016/j.arth.2014.09.022

[18] Guyen O, Pibarot V, Vaz G, et al. (2007) Unconstrained tripolar implants for primary total hip arthroplasty in patients at risk for dislocation. J Arthroplasty 22, 849–858. 17826276 10.1016/j.arth.2006.11.014

[19] Prudhon JL (2011) Dual-mobility cup and cemented femoral component: 6 year follow-up results. Hip Int 21, 713–717. 22117262 10.5301/HIP.2011.8846

[20] Bouchet R, Mercier N, Saragaglia D (2011) Posterior approach and dislocation rate: a 213 total hip replacements case-control study comparing the dual mobility cup with a conventional 28-mm metal head/polyethylene prosthesis. Orthop Traumatol Surg Res 97, 2–7. 21177149 10.1016/j.otsr.2010.07.008

[21] Boyer B, Neri T, Geringer J, et al. (2017) Long-term wear of dual mobility total hip replacement cups: explant study. Int Orthop 42, 41–47. 28577036 10.1007/s00264-017-3525-z

[22] Callaghan JJ, Forest EE, Sporer SM, et al. (1997) Total hip arthroplasty in the young adult. Clin Orthop Relat Res 257–262. 9372776

[23] Dangin A, Boulat S, Farizon F, Philippot R (2016) Prevention of dislocation risk during hip revision surgery with the dual mobility concept; study of a new generation of dual mobility cups. Surg Technol Int 29, 314–319. 27728956

[24] Philippot R, Baulot E, Vermorel P-H, et al. (2019) Outcomes of Augmented Dual Mobility Acetabular Cups. Surg Technol Int 35, 274–279. 31373376

[25] Wegrzyn J, Pibarot V, Jacquel A, et al. (2014) Acetabular reconstruction using a Kerboull cross-plate, structural allograft and cemented dual-mobility cup in revision THA at a minimum 5-year follow-up. J Arthroplasty 29, 432–437. 23849510 10.1016/j.arth.2013.05.030

[26] Schneider L, Philippot R, Boyer B, Farizon F (2011) Revision total hip arthroplasty using a reconstruction cage device and a cemented dual mobility cup. Orthop Traumatol Surg Res 97, 807–813. 22119512 10.1016/j.otsr.2011.09.010

[27] Sayac G, Neri T, Schneider L, et al. (2020) Low revision rates at more than 10 years for dual-mobility cups cemented into cages in complex revision total hip arthroplasty. J Arthroplasty 35, 513–519. 31543421 10.1016/j.arth.2019.08.058

[28] Neri T, Boyer B, Geringer J, et al. (2018) Intraprosthetic dislocation of dual mobility total hip arthroplasty: still occurring? Int Orthop 43, 1097–1105. 30027352 10.1007/s00264-018-4054-0

[29] Malatray M, Roux J-P, Gunst S, et al. (2017) Highly crosslinked polyethylene: a safe alternative to conventional polyethylene for dual mobility cup mobile component. A biomechanical validation. Int Orthop 41, 507–512. 27837329 10.1007/s00264-016-3334-9

[30] Di Laura A, Hothi HS, Henckel J, et al. (2017) Retrieval evidence of impingement at the third articulation in contemporary dual mobility cups for total hip arthroplasty. Int Orthop 41, 2495–2501. 28578471 10.1007/s00264-017-3523-1

[31] Tsukada S, Wakui M (2015) Lower dislocation rate following total hip arthroplasty via direct anterior approach than via posterior approach: five-year-average follow-up results. Open Orthop J 9, 157–162. 26157532 10.2174/1874325001509010157 PMC4483535

[32] Sariali E, Leonard P, Mamoudy P (2008) Dislocation after total hip arthroplasty using Hueter anterior approach. J Arthroplasty 23, 266–272. 18280423 10.1016/j.arth.2007.04.003

[33] Poehling-Monaghan KL, Kamath AF, Taunton MJ, Pagnano MW (2015) Direct anterior versus miniposterior THA with the same advanced perioperative protocols: surprising early clinical results. Clin Orthop Relat Res 473, 623–631. 25082624 10.1007/s11999-014-3827-z PMC4294903

[34] Batailler C, Fary C, Batailler P, et al. (2017) Total hip arthroplasty using direct anterior approach and dual mobility cup: safe and efficient strategy against post-operative dislocation. Int Orthop 41, 499–506. 27853816 10.1007/s00264-016-3333-x

[35] Judet J, Judet H (1985) Anterior approach in total hip arthroplasty. Presse Med 14, 1031–1033. 3158949

[36] Philippeau J-M, Durand J-M, Carret J-P, et al. (2010) Dual mobility design use in preventing total hip replacement dislocation following tumor resection. Orthop Traumatol Surg Res 96, 2–8. 20170850 10.1016/j.rcot.2009.12.011

[37] Klement MR, Hubbard EW, Wellman SS, Lachiewicz PF (2017) Acute intraprosthetic dissociation of a dual-mobility hip in the united states. Am J Orthop 46, E154–E159. 28666047

[38] Odland AN, Sierra RJ (2014) Intraprosthetic dislocation of a contemporary dual-mobility design used during conversion THA. Orthopedics 37, e1124–1128. 25437088 10.3928/01477447-20141124-90

[39] Waddell BS, De Martino I, Sculco T, Sculco P (2016) Total hip arthroplasty dislocations are more complex than they appear: a case report of intraprosthetic dislocation of an anatomic dual-mobility implant after closed reduction. Ochsner J 16, 185–190. 27303232 PMC4896666

[40] Tyagi V, Akinbo O (2017) Early intraprosthetic dislocation of a dual mobility acetabular construct after total hip arthroplasty. J Orthop Case Rep 7, 21–24. 10.13107/jocr.2250-0685.732 PMC5553828 28819595

[41] Loubignac F, Felts E, Allal R (2012) Early intraprosthetic dislocation of a total hip replacement with dual mobility socket: clinical presentation and update review. Eur J Orthop Surg Traumatol 22 (Suppl 1): 85–87. 26662755 10.1007/s00590-011-0906-7

[42] De Martino I, Triantafyllopoulos GK, Sculco PK, Sculco TP (2014) Dual mobility cups in total hip arthroplasty. World J Orthop 5, 180–187. 25035820 10.5312/wjo.v5.i3.180 PMC4095010

[43] Cvetanovich GL, Fillingham YA, Della Valle CJ, Sporer SM (2015) Intraprosthetic dislocation of dual-mobility bearings associated with closed reduction: a report of two cases. JBJS Case Connect 5, e26. 10.2106/JBJS.CC.N.00137 29252732

[44] Whiteside LA (2012) Surgical technique: transfer of the anterior portion of the gluteus maximus muscle for abductor deficiency of the hip. Clin Orthop Relat Res 470, 503–510. 21796476 10.1007/s11999-011-1975-y PMC3254750

[45] Tsang S-TJ, Ting J, Simpson AHRW, Gaston P (2017) Outcomes following debridement, antibiotics and implant retention in the management of periprosthetic infections of the hip: a review of cohort studies. Bone Joint J 99-B, 1458–1466. 29092984 10.1302/0301-620X.99B11.BJJ-2017-0088.R1

[46] Aldinger PR, Jung AW, Pritsch M, et al. (2009) Uncemented grit-blasted straight tapered titanium stems in patients younger than fifty-five years of age. Fifteen to twenty-year results. J Bone Joint Surg Am 91, 1432–1439. 19487522 10.2106/JBJS.H.00297

[47] Kim Y-H, Park J-W, Park J-S (2014) The 27 to 29-year outcomes of the PCA total hip arthroplasty in patients younger than 50 years old. J Arthroplasty 29, 2256–2261. 24636903 10.1016/j.arth.2014.02.011

[48] Yoon TR, Rowe S-M, Kim M-S, et al. (2008) Fifteen- to 20-year results of uncemented tapered fully porous-coated cobalt-chrome stems. Int Orthop 32, 317–323. 17323091 10.1007/s00264-007-0337-6 PMC2323415

[49] Grant P, Nordsletten L (2004) Total hip arthroplasty with the Lord prosthesis. A long-term follow-up study. J Bone Joint Surg Am 86-A, 2636–2641. 10.2106/00004623-200412000-00008 15590847

[50] Bojescul JA, Xenos JS, Callaghan JJ, Savory CG (2003) Results of porous-coated anatomic total hip arthroplasty without cement at fifteen years: a concise follow-up of a previous report. J Bone Joint Surg Am 85-A, 1079–1083. 10.2106/00004623-200306000-00015 12784006

[51] Della Valle CJ, Mesko NW, Quigley L, et al. (2009) Primary total hip arthroplasty with a porous-coated acetabular component. A concise follow-up, at a minimum of twenty years, of previous reports. J Bone Joint Surg Am 91, 1130–1135. 19411461 10.2106/JBJS.H.00168

[52] Wroblewski BM (1986) 15–21-year results of the Charnley low-friction arthroplasty. Clin Orthop Relat Res (211) 30–35. 3769266

[53] Berry DJ, Harmsen WS, Cabanela ME, Morrey BF (2002) Twenty-five-year survivorship of two thousand consecutive primary Charnley total hip replacements: factors affecting survivorship of acetabular and femoral components. J Bone Joint Surg Am 84-A, 171–177. 10.2106/00004623-200202000-00002 11861721

[54] Callaghan JJ, Templeton JE, Liu SS, et al. (2004) Results of Charnley total hip arthroplasty at a minimum of thirty years. A concise follow-up of a previous report. J Bone Joint Surg Am 86-A, 690–695. 10.2106/00004623-200404000-00004 15069131

[55] Vigdorchik JM, D’Apuzzo MR, Markel DC, et al. (2015) Lack of early dislocation following total hip arthroplasty with a new dual mobility acetabular design. Hip Int 25, 34–38. 25655740 10.5301/hipint.5000186

[56] Jauregui JJ, Pierce TP, Elmallah RK, et al. (2016) Dual mobility cups: an effective prosthesis in revision total hip arthroplasties for preventing dislocations. Hip Int 26, 57–61. 26391258 10.5301/hipint.5000295

[57] Lecuire F, Benareau I, Rubini J, Basso M (2004) Intra-prosthetic dislocation of the Bousquet dual mobility socket. Rev Chir Orthop Reparatrice Appar Mot 90, 249–255. 15211274 10.1016/s0035-1040(04)70101-4

[58] Philippot R, Boyer B, Farizon F (2013) Intraprosthetic dislocation: a specific complication of the dual-mobility system. Clin Orthop Relat Res 471, 965–970. 23054529 10.1007/s11999-012-2639-2 PMC3563829

[59] Wegrzyn J, Malatray M, Pibarot V, et al. (2020) Is isolated mobile component exchange an option in the management of intraprosthetic dislocation of a dual mobility cup? Clin Orthop Relat Res 478, 279–287. 31794492 10.1097/CORR.0000000000001055 PMC7438138

[60] Hernigou P, Dubory A, Potage D, et al. (2017) Dual-mobility arthroplasty failure: a rationale review of causes and technical considerations for revision. Int Orthop 41, 481–490. 27872981 10.1007/s00264-016-3328-7

